# Supplementary material for: Patterns of Intron Gain and Loss in Fungi
Source: PLoS Biol. 2004 Nov 30;2(12):e422. doi: 10.1371/journal.pbio.0020422 (PMC532390; doi:10.1371/journal.pbio.0020422)
Supplement: Table S1 — Also available at http://genes.mit.edu/NielsenEtAl/. (4.3 MB ZIP). [file pbio.0020422.st001.zip › NielsenEtAl/html/643.html]

AN4017.1.NCU05276.1.MG06045.1.FG01957.1


```
 CLUSTAL W (1.82) Multiple Sequence Alignments - Introns Inserted


Sequence 1: NCU05276.1	314 aa
Sequence 2: MG06045.1	281 aa
Sequence 3: FG01957.1	279 aa
Sequence 4: AN4017.1	301 aa
Alignment Length: 316 aa
Number Identitical Residues: 149 aa
Alignment Score (without introns) 7235


MG06045.1 	MSGPGYDAVVDIDDE0GD-LGHTDLQDDLEFHNST~FSETTPGARK---------PGAVG
NCU05276.1	MSRSGYDAVVDVDDE0GD-LGHTDLQEDLEFHQSN~FSESSPLGRNGTKSTQHTNTTSTS
FG01957.1 	MSGSGYDAVVDVDDE0GD-LGHTDLQEDLEFHTSN~FNDTNPNTRK-------------P
AN4017.1  	MSNQGYDVVVDVDAE0GDDLGHTDLQEDLEFHPSN1FENDQRNAKS--------HPDNAA
          	**  ***.***:* * **.*******:***** *. *.:     :.         .    

MG06045.1 	GAGSSGLPLPATASTVSG-------KRYLWTVSYYAQFFDVDTSAVLSRCWAALYPRANF
NCU05276.1	SLGGGLLPAPVTAARTSSNSGGGSSKRFLWTLSFYAQFFDVDTSSVLQRCWAALYPRANF
FG01957.1 	PSGGSGLPAPATASSGSG-----SSKRFLWSMNFYAQFFDVDTSAVLSRCWAALFPRANF
AN4017.1  	FLGSGGGSSSRRNRSPGG----TPTKHAWWSIHYYEQYFDVDTNEVLRRCVASVYPRSNF
          	  *..  . .      ..     .:*:  *:: :* *:*****. ** ** *:::**:**

MG06045.1 	LDVLEGNPDLYGPFWIATTVIFILFIGGTISQYLATRGTTEAFVYDFTLLSG1AAGLIYG
NCU05276.1	LDVLEGNPDLYGPFWIATTVVLILFLGGTISDYLAQTG-KEAFAYDFRLLSG1AAGLIYG
FG01957.1 	LDVLEGNPDLYGPFWIATTVVLILFLGGTISQYMSDTG-KGPFLYDFKLLSG1AAGLIYG
AN4017.1  	LDVLEGNADLYGPFWIATTVIVILFLTGTISQWLSNND-KEHFKYDFTLLSG~AAGLVYG
          	*******.************:.***: ****::::  . .  * *** **** ****:**

MG06045.1 	YTLFLPVVLFLALRYFGSESANLLECWALYGYANLIWIPVALISWSQIDILNFVFVAVGF
NCU05276.1	YTFVLPVALYLALRYFGSESANLLECWALYGYSNLIWIPVALISWSPITILNWVFVGVGF
FG01957.1 	YTLFIPMGLFLALRYFGSESANLLECWALYGYSNLIWIPVALISSSPISILNWVFVGVGF
AN4017.1  	YTFALPIALWGALKWFGSSTADLVECWALYGYSNLVWIAVALVSWSPLTALNWALVGVGF
          	**: :*: *: **::***.:*:*:********:**:**.***:* * :  **:.:*.***

MG06045.1 	GYSVAFLLRNLYPVLSATDRQTSKILLIVVVALHFALAVTIKILFFA~HHSPVAK-----
NCU05276.1	GVSVAFLLRNLYPVLSATDRQTSKVLLFVVVALHFGLALAIKVLFFA~HGSPALKDGPKG
FG01957.1 	GISVAFLLRNLYPVLSATDRQVSKVLLVVVVLLHAGLSLAIKILFFA~HGSPVSS-----
AN4017.1  	AWTVFFLLRNLYPVLSATDAKASRALLILVIVLHAGFAIAIKILFFA2HGSPVSKKGDSE
          	. :* ************** :.*: **.:*: ** .::::**:**** * **. ... . 

MG06045.1 	---GDGKAQT----------
NCU05276.1	AAPGGGDAKPADPATMMMMF
FG01957.1 	---TPDKSKGD---------
AN4017.1  	KGRDDDKDDHDDHDKRMF--
          	 .   .. .  .  .
```
